# Improved analysis of bacterial CGH data beyond the log-ratio paradigm

**DOI:** 10.1186/1471-2105-10-91

**Published:** 2009-03-19

**Authors:** Lars Snipen, Otto L Nyquist, Margrete Solheim, Ågot Aakra, Ingolf F Nes

**Affiliations:** 1 Biostatistics, Department of Chemistry, Biotechnology and Food Sciences, Norwegian University of Life Sciences, Ås, Norway; 2 Laboratory of Microbial Gene Technology, Department of Chemistry, Biotechnology and Food Sciences, Norwegian University of Life Sciences, Ås, Norway

## Abstract

**Background:**

Existing methods for analyzing bacterial CGH data from two-color arrays are based on log-ratios only, a paradigm inherited from expression studies. We propose an alternative approach, where microarray signals are used in a different way and sequence identity is predicted using a supervised learning approach.

**Results:**

A data set containing 32 hybridizations of sequenced versus sequenced genomes have been used to test and compare methods. A ROC-analysis has been performed to illustrate the ability to rank probes with respect to Present/Absent calls. Classification into Present and Absent is compared with that of a gaussian mixture model.

**Conclusion:**

The results indicate our proposed method is an improvement of existing methods with respect to ranking and classification of probes, especially for multi-genome arrays.

## Background

Microarray based comparative genomic hybridizations (CGH) is a tool for rapid investigation of the genetic content of bacteria. The technique is used for comparative genomic studies as well as screening for virulence factors or other genomic features of interest in a population [1-3]. The basic idea behind the technology is to construct microarrays from sequenced and annotated genomes, and then hybridize genomic DNA from other sources to these arrays to detect similarities and differences in genomic content. For two-color arrays DNA from some sampled genome is labeled and hybridized against labeled DNA from a reference. This reference is typically genomic DNA from one or several fully sequenced genomes, usually those from which the array was constructed.

The results obtained from such experiments can be seen as projections of the genomes in question onto the sequence space spanned by the microarray probe sequences. This probe space may vary in size, representing only a set of selected genomic features all the way up to pan-genomes. Probes may be short or long oligonucleotides, or PCR products, and we will in this paper only consider cases where the probe sequences are known exactly.

The data from these experiments are qualitatively different from those obtained in gene expression studies, where signal intensities must be seen as a continuum due to the dynamic abundance of mRNA. In bacterial CGH (bCGH) differences in signal intensities are predominately due to differences in sequence composition, copy number abberations are few and give smaller signal fluctuations. For this reason bCGH signals tend to behave more like a categorical variable with two possible outcomes, usually denoted Present and Absent. A strong signal, corresponding to Present, means the corresponding probe sequence is found, with sufficient similarity to yield hybridization, in the investigated genome. A weak signal means a too small part of the probe sequence is found in the genome to give hybridization, and the probe is called Absent.

Some methods to analyze bCGH data of this type have been proposed, and some of them are reviewed and tested in a recent publication by [4]. Most of these methods base their results on the log-ratio of signals, which is a standard adopted from the analysis of expression data. We will in this paper propose a new strategy for analyzing bCGH data, that does not rely on log-ratios, which we believe is a misleading paradigm for this type of data. Also, some previously proposed methods utilizing more than just log-ratios, like [5-7], are all unsupervised methods, not taking into account the sequence information from the reference genomes. In our approach this information is also included to aid the analysis. In the analysis of two-color microarray data demonstrated in this paper, we treat the array signals separately, almost as two single-color arrays, hence the method could easily be used for data from this technology as well. We test our method on data from *S. aureus *and *E. faecalis*, and compare our results to those achieved by other effective methods.

## Methods

### Sequence identity

Any bCGH experiment starts by performing alignments of every array probe sequence against the fully sequenced reference genomes to establish which probes are present and absent in these genomes. We use the term R-genome for a reference genome. We define the *identity *between probe and an R-genome as the number of identical bases in the best local alignment between them divided by the probe length to obtain a value between 0 and 1. We call this quantity *Rb*_*ij *_for probe *i *against the R-genome in hybridization *j*. If *Rb*_*ij *_= 1 it means an exact copy of the probe sequence is found in the R-genome, while if probe *i *has no significant hits in R-genome *j *we set *Rb*_*ij *_= 0 even if all probes will of course have some very short subsequences in common with any genome.

The categorical response Present or Absent is coded as 1 or 0, respectively. This require, however, that intermediate *Rb*-values must be rounded to either 1 or 0, i.e. we need some *a priori *threshold that specify the sequence identity needed to be Present. Our analysis approach does not require categorical responses, and intermediate *Rb*-values can be used as is. However, if the ultimate goal is to classify between Present and Absent, the analysis is usually favored by having only 1 and 0 as responses from the start.

A sampled, un-sequenced, genome we call a sample-genome or S-genome. Corresponding to *Rb*_*ij *_for the R-genome, we also have a similar *Sb*_*ij *_for the S-genome. The motivation behind the entire bCGH experiment is to say something about this *Sb*_*ij*_, i.e. the sequence identity between probe *i *and the S-genome in hybridization *j*.

### Preprocessing

For each array in the experiment, we assume background correction and within-array normalization has been done. We have employed standard methods in the LIMMA package [8] in R [9], available from the Bioconductor [10]. Normalization of CGH arrays has recently been discussed by [11] and [12], and nothing in our downstream analysis prevent the use of these or other approaches.

The flagging of low quality spots should be done very careful for bCGH analyses. In standard procedures for expression data, spots with low signals are removed. For bCGH data these spots turn out informative, because they span the range of array signals. Especially negative control probes, e.g. spots with alien or no DNA, are important since they carry information about which signals to expect when no hybridization takes place.

On microbial arrays probes are usually spotted multiple times (replicates). We will only consider the median value of these replicates on each array, but the number of replicates for each probe is kept as a weight in the final prediction, i.e. probes with more replicates have larger impact. Let  and  be the median preprocessed log-transformed signals from the R- and S-genome channel for probe *i *in hybridization *j*.

In most cases a bCGH experiment will consist of a batch of several hybridizations to be analyzed simultaneously. For our downstream analysis some between-array normalization within this batch is beneficial. Let *I*_*j*0 _= {*i*|*Rb*_*ij *_< 0.1}, i.e. the set of probes with R-genome sequence identity less than 0.1. Also, let *I*_*j*1 _= {*i*|*Rb*_*ij *_> 0.9}. Let *Ra*_*j*0 _and *Ra*_*j*1 _be the median of the *Ra*_*ij *_values for the probes in *I*_*j*0 _and *I*_*j*1_, respectively. Then the between-array normalized R-signal is

(1)

**Figure 1:**
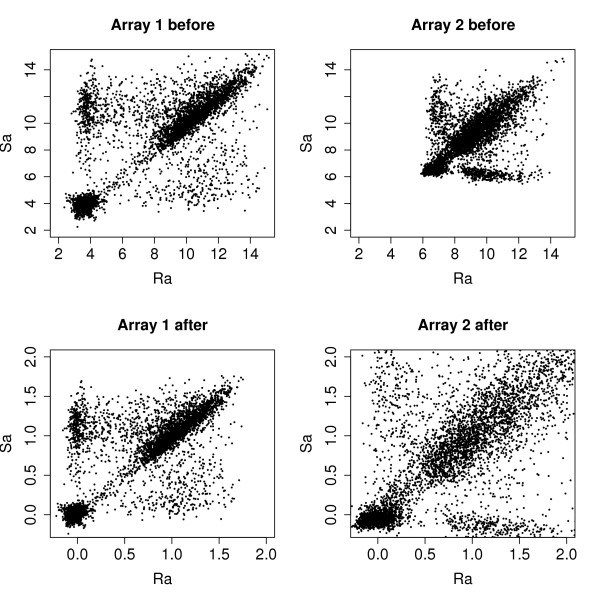
**Effect of between-array normalization**. Plots show log-transformed array signal from S-genome (*Sa*) against R-genome (*Ra*) for two arrays before (upper panels) and after (lower panels) between-array normalization.

The signals from the S-genome channel is treated the same way, only replacing  with , obtaining the normalized signal *Sa*_*ij*_. Notice that this procedure requires a significant number of probes to have low (less than 0.1) sequence identity with the R-genome, i.e. negative control probes are essential here. The effect of this normalization can be seen in Figure 1.

### Probe bias

Given a sequence identity *Rb*_*ij*_, the corresponding array signals *Ra*_*ij *_will in general correlate in a positive way, i.e. stronger sequence identity yields stronger array signal, and a similar relation we assume also holds between *Sa*_*ij *_and the the unknown *Sb*_*ij*_. However, probes with similar *Rb*-value may show consistently different *Ra*-values. This reflects a variable signal potential for the different probes due to sequence composition and/or bias during construction of the arrays. We refer to this as the probe bias. The same probe bias we assume is also present in the relation between *Sb*_*ij *_and *Sa*_*ij*_.

The *Rb*-values take on *L *discrete values between 0 and 1, and consider subsets of probes with similar *Rb*-value, i.e. ℐ_*l *_= {*i*|*Rb*_*ij *_= *l*} for *l *= 0,...,1. We assume for all *i *∈ ℐ_*l *_and hybridization *j *the linear model

(2) *Ra*_*ij *_= *μ*_*lj *_+ *B*_*ij*_

where *μ*_*lj *_is the unconditional expected array signal at *Rb*-value *l *and *B*_*ij *_is the probe bias for probe *i *in hybridization *j*. From this we get estimates of the probe bias for each hybridization .

For probe *i *we can get a pooled estimate of probe bias by averaging over the *J *hybridizations, i.e.

(3)

If arrays are similar with respect to this bias the estimate  is less variable than . On the other hand, if some arrays differ substantially with respect to this bias, the pooled estimate is poor. To cope with all situations we introduce a weight *ω *∈ [0,1] and use as the final estimate of probe bias

(4)

Choosing *ω *close to 1 means information is 'borrowed' across hybridizations.

### Predicting sequence identity

The basic idea is, for each array, to fit a function that describes how *Rb*-values depend on bias-corrected *Ra*-values, and then use the same function to predict *Sb*-values from bias-corrected *Sa*-values.

First, we assume there is a function *f*_*j *_for hybridization *j *such that

(5)

We will make few assumptions about the shape of the function *f*_*j*_, but we will require it to be monotonously increasing, since an increased array signal should always indicate stronger sequence identity.

We have chosen to estimate *f*_*j *_by a weighted running mean, where probes are weighted by their number of within-array replicates. For notational simplicity, let *x*_*ij *_= *Ra*_*ij *_- . The range of the function is divided into *N *equally spaced *knots*, *x*_1_,...,*x*_*N*_, and let *D *be the width between two knots. For knot *n*, let *C*_*n *_be the data subset {*x*_*ij*_, *Rb*_*ij*_} whose value of *x*_*ij *_falls within *x*_*n *_± 3*D*/2. Finding *f*_*j*_(*x*_1_),...,*f*_*j*_(*x*_*N*_) leads to the constrained optimization problem



This problem can be solved by first computing the unconstrained optimum (weighted running mean), and then resolving the violated constraints in a recursive way. If the initial estimate of *f*_*j*_(*x*_*n*+1_) is smaller than that of *f*_*j*_(*x*_*n*_), both are replaced by the weighted average of them, weighted by the number of data points behind each initial estimate. This may again violate the constraints on the estimates of *f*_*j*_(*x*_*n*+2_) and/or *f*_*j*_(*x*_*n*-1_), and hence the recursion.

**Figure 2:**
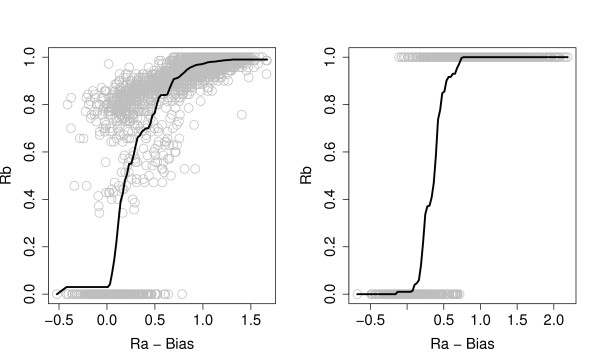
**Predicting sequence identity**. An illustration of how an estimated function  maps bias-corrected normalized array signal *Raij *-  onto sequence identity *Rb*_*ij*_. Gray circles are data, black curve indicate the function . In the left panel the sequence identities are used as is, while in the right panel all *Rb *values above 0.7 have been set to 1.0, and all below 0.7 to 0.0, corresponding to Present and Absent, respectively.

Given the estimates of *f*_*j*_(*x*_1_),...,*f*_*j*_(*x*_*N*_) the estimated function value at any point within the range is found by linear interpolation between the knots. Let  denote this estimated function for array *j*. Figure 2 illustrates how  fits a typical data set.

For the given S-genome in hybridization *j*, the prediction of the sequence identity for probe *i *is now given as

(6)

It is not uncommon to repeat experiments, i.e. hybridize the same S-genome to several arrays. In this case it is natural to first analyze each array separately, obtain predictions from each array, and in the end average these  for each S-genome. A description of uncertainty in the prediction is best achieved by constructing a confidence interval for *Sb*_*ij*_. Since this variable is trapped between 0 and 1 it seems reasonable to avoid inference based on specific distributions, and instead rely on some non-parametric approach. In case of a categorical response (Present/Absent), majority vote should be used instead of average, and statements concerning uncertainty should be put forward as some estimate of posterior probability of Present. The proportion of Present-votes for each probe is the maximum likelihood estimate of this probability, assuming the repeated experiments are independent.

### Data

In order to test methods we performed bCGH experiments using only sequenced genomes, i.e. the *Sb*-values are, contrary to a real situation, all known. Two different arrays were used, one representing 6 genomes of *Staphylococcus aureus *available from J. Craig Venter Institute [13] (JCVI), and one representing the genome of *Enterococcus faecalis *strain V583. In both cases probes are 70-mer oligonucleotides. The *S. aureus *array contains 5057 different probes spotted six times, where 4515 are ordinary probes representing genomes, and the remaining 542 negative control probes include various alien DNA and the 'empty probe' (no DNA). The *E. faecalis *array contains 3218 probes representing genes in the genome of V583, 10 probes representing the enterococcal pathogenicity island of strain MMH594 and 15 negative controls, giving a total of 3243 probes, spotted three times each.

**Figure 3:**
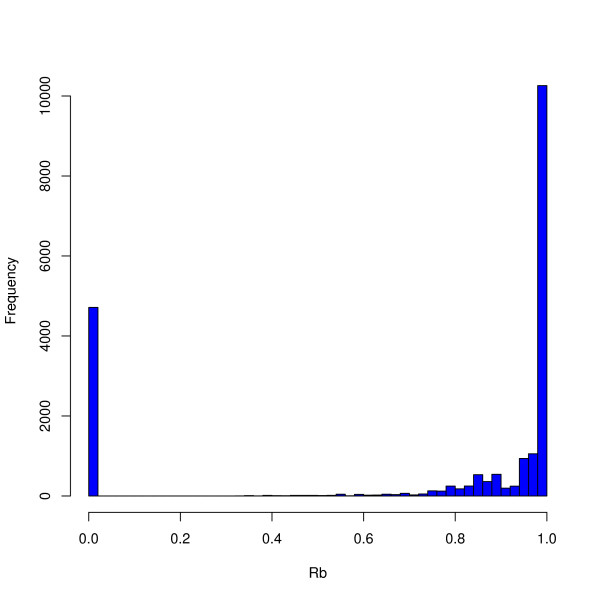
**Distribution of *R*_*b *_values**. The histogram shows the distribution of *Rb*-values when BLASTing the probe sequences against four of the genomes they are designed to represent. A majority of alignments show either *Rb *= 0 or *Rb *= 1, but a large proportion of probes also have 0.7 <*Rb *< 1.0. By choosing the threshold between Present and Absent at 0.7 these probes are defined as Present.

Experiments were conducted using the *S. auerus *strains COL, N315, Mu50, NCTC8325 and RF122 and *E. faecalis *strains V583 and OG1RF, whose genome sequences are available at NCBI [14]. For the *S. aureus *experiments seven different pairs of genomes were selected for hybridization, and for each pair a dye-swap was performed. For each of these 14 hybridizations both genomes involved can play the role as R-genome and S-genome, hence there are altogether 28 different *S. aureus *data sets where we can compare predicted and true sequence identity. Two hybridizations of V583 versus OG1RF were conducted (dye swap), and again both genomes can play the role as R-genome and S-genome, giving 4 additional *E. faecalis *data sets. In order to compare our method against other methods we use a categorical response, i.e. each probe is classified as Present (1) or Absent (0). This means we have assigned a threshold to the *Rb*- and *Sb*-values in order to round each value to 1 or 0. We have used the threshold 0.7 (70% identity), i.e. an *Rb*- or *Sb*-value above 0.7 corresponds to Present and is rounded to 1 and values below 0.7 is rounded to 0. The threshold is chosen on the basis of the histogram in Figure 3. The *S. aureus *arrays contain probes representing genes in 6 different strains. By BLASTing the probe sequences against the genome sequences of these strains, the identities distribute as indicated in Figure 3. Thus, it seems that probes matching with approximately 70% identity or more are considered Present in the genome by JCVI who designed the arrays. This also corresponds well with our experience regarding the degree of match giving hybridizations. This threshold will in general depend on array design and hybridization conditions, and a proper value must be decided upon for each experiment separately. Our method is independent of this choice as long as it is a reasonable value for the experiments analyzed. Table 1 show the percent of truly Present/Absent probes in each of the genomes using our probe set and threshold.

**Table 1:** Genomes and microarrays

Genome	Size (Mb)	Present	Absent
*S. aureus *COL	2.81	74%	26%
*S. aureus *N315	2.84	74%	26%
*S. aureus *Mu50	2.90	76%	24%
*S. aureus *NCTC8325	2.82	74%	26%
*S. aureus *RF122	2.74	70%	30%
*E. faecalis *V583	3.36	99%	1%
*E. faecalis *OG1RF	2.73	73%	27%

Overview of the genomes used in the test data sets. The Present/Absent numbers indicate the percentage of probes on the arrays having sequence identity above (Present) or below (Absent) the threshold of *Rb *= 0.7. Percentages refer to the total number of probes, i.e. 5057 on the *S. aureus *array and 3243 on the *E. faecalis *array.

As previously mentioned, we advocate a weak flagging of array spots during the preprocessing of the data. This means only truly damaged spots should be flagged, and spots with weak signals or negative controls, should be part of the data set through the entire analysis. When comparing our proposed method against other approaches, we used both 'hard' and 'weak' flagging of spots to illustrate the differences between these strategies. By 'hard' flagging we mean removing all negative controls as well as all spots flagged by the image analysis software, i.e. in our case all spots with negative flag value from GenePix. In the 'weak' flagging only manually discarded spots were removed, i.e. only spots with flag value -100 from GenePix.

## Results

Our proposed method predicts probe sequence similarity to a sampled genome based on a biased-corrected array signal. Based on observed array signal and probe sequence similarities to the reference genome, we estimate a probe bias for each probe. Then, correcting for this probe bias, we fit a non-parametric function describing the relation between array signal and probe sequence similarity for the reference genome. Finally, we use this function to predict probe sequence similarity from observed array signals for the sampled genome. If a categorical response (Present or Absent) is desired this is coded as Present = 1 and Absent = 0. Comparison to other approaches are here made on data sets where true sequence similarities (Present/Absent status) are known.

### ROC-analysis

**Figure 4:**
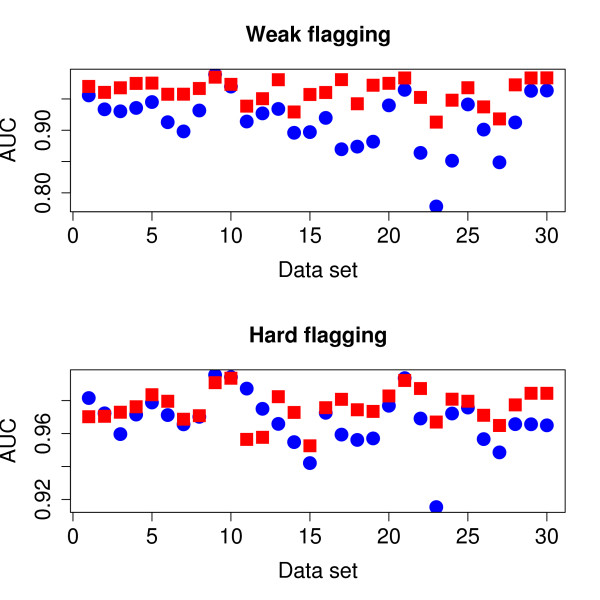
**ROC analysis**. The plots show AUC statistics for the 28 *S. aureus *data sets and 2 of the 4 *E. faecalis *data sets. Only ordinary probes (negative control probes excluded) are ranked either by log-ratio (blue dots) or bias-corrected S-signal (red squares). In the upper panel we have used weak, and in the lower panel hard flagging, i.e. in the lower panel fewer probes are ranked.

Most bCGH analyses are based on the ranking of probes according to log-ratios. In our approach the corresponding ranking is according to predicted sequence similarity. The potential for correct classification was examined by ranking all ordinary probes by both criteria, and the Area Under Curve (AUC) statistic from a ROC-analysis [15] was computed for the data sets. Under the hard flagging regime, two of the *E. faecalis *data sets completely lacked absent probes, and hence no AUC-values could be computed for these data sets. Thus, only 2 of the 4 *E. faecalis *data sets were included in the ROC-analysis. Figure 4 shows the AUC-values for both ranking criteria. An AUC-value of 1.0 means perfect separation of classes, while a value close to 0.5 means ranking is completely random, i.e. both classes are mixed in the ranked list. In this analysis we used the weight *ω *= 0.75 to compute the probe bias effect. Other choices of these weights produced very similar AUC-values, and did not alter the big picture.

### Effect of bias weight *ω*

**Figure 5:**
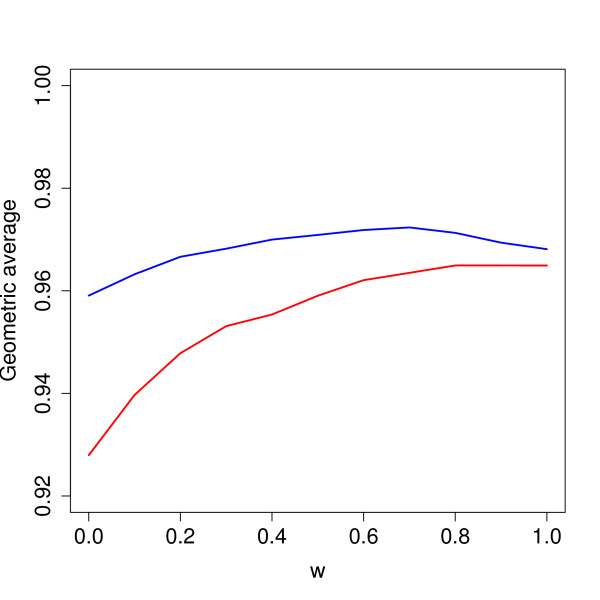
**Optimal choice of *ω***. The curves indicate geometric average of sensitivity and specificity after classification for various combinations of the weight *ω *for the *S. aureus *(lower, red) and *E. faecalis *(upper, blue) arrays. A larger geometric average means better classification, and a value of 1.0 means perfect separation. The geometric average is first computed for every data set separately, and then averaged over the 28 *S. aureus *and 4 *E. faecalis *data sets.

Our proposed method depends on the choice of the weight *ω *from (4). A weight close to 1 means information is borrowed between arrays when it comes to estimating the probe bias. To get an impression of the effect of this constant, we varied it systematically over the interval [0, 1], and for each weight classified all probes in all data sets. For each data set we computed the classification error as the geometric average [16]. This is the square root of the product of sensitivity (probability of classifying as Present when truly Present) and specificity (probability of classifying as Absent when truly Absent). Figure 5 illustrate how the geometric average varies for different choices of *ω *over the *S. aureus *and *E. faecalis *data sets.

### Comparing classification results

In the review by [4], the best classification was obtained by fitting a gaussian mixture model to the log-ratio distribution on each array separately. Using a two-component mixture, interpreted as the Present and Absent component, probes are then classified into Present/Absent based on the posterior probabilities [17]. Hence, we have chosen this as a standard method for comparison. We classified probes in all 32 data sets with a log-ratio based mixture model as well as our proposed method, which we here refer to as bias-corrected S-signal prediction (BCSP). Log-ratios were within-array normalized using the LIMMA-package, as described in the Methods section. For each data set, and each method, we computed the sensitivity, specificity, positive predicted value (PPV) and negative predicted value (NPV). PPV is the estimate of the probability of a probe being truly Present when classified as Present, and NPV similar for Absent. The exercise was done for both hard and weak flagging. In all cases the negative control probes were removed before classification error was computed, i.e. classification quality was only measured on ordinary probes. Table 2 summarize the results.

**Table 2:** Classification results

Array	Flagging	# probes	Method	Sens.	Spec.	PPV	NPV
*S. aureus*	Weak	4515	BCSP	0.968	0.938	0.987	0.861
			Mix.mod.	0.945	0.901	0.979	0.776
			p-value	4.1·10^-7^	7.4·10^-4^	1.7·10^-4^	2.5·10^-7^
	
	Hard	3539	BCSP	0.977	0.864	0.989	0.754
			Mix.mod.	0.960	0.825	0.985	0.607
			p-value	3.2·10^-8^	0.02	0.003	3.7·10^-8^

*E. faecalis*	Weak	3228	BCSP	0.989	0.953	0.983	0.560
			Mix.mod.	0.946	0.890	0.989	0.458
	
	Hard	3145	BCSP	0.992	0.0	0.869	0.0
			Mix.mod.	0.955	0.474	0.990	0.409

Classification of probes into Present/Absent was done after both weak and hard flagging, see text for details, and using both our bias-corrected S-signal prediction method (BCSP) and the gaussian mixture model method. The number of probes indicate the median number of ordinary probes in a data set after flagging. All the results shown (Sens., Spec., PPV and NPV) are averages over the 28 *S. aureus *or 4 *E. faecalis *data sets. The p-values listed for the *S. aureus *results are the outcome of a Wilcoxon signrank test of wether the BCSP-method is a significant improvement of the mixture model method. A smaller p-value indicate a significant improvement. For the *E. faecalis *results all p-values were large (> 0.10) and they are not listed.

### Prediction error

**Figure 6:**
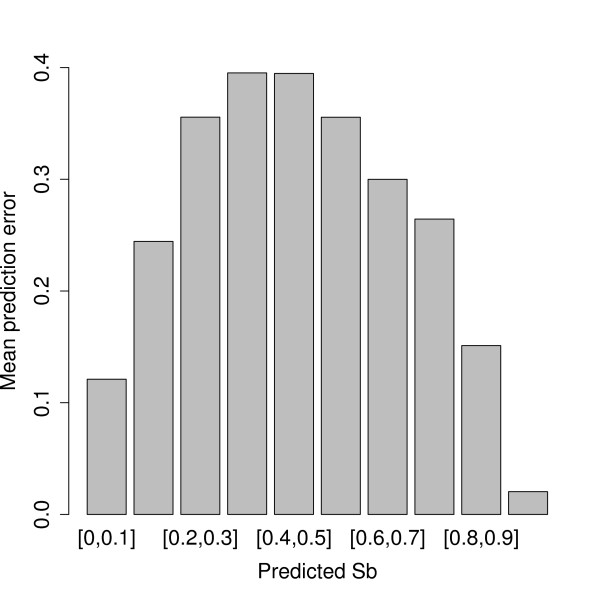
**Prediction error**. The distribution of absolute error of prediction |*Sb*_*ij *_- | over predicted sequence identity  for all data sets. Each bar is the average prediction error in the corresponding interval.

Using our BCSP method, we can in principle predict the degree of Presence of a given probe. In order to do this *Rb*-values should not be rounded to 0 or 1, but used as is, as illustrated in the left panel of Figure 2. However, since the large majority of probes are either completely present or absent, predicting an intermediate sequence identity is usually a sign of uncertainty of the probes actual status. This is reflected in Figure 6, where we have indicated the average absolute error | - *Sb*_*ij*_| for the different predicted values .

## Discussion

There is at present no standard approach for analyzing bacterial CGH data, and the methods reviewed by [4] are only a selection of approaches employed in recent bCGH-publications, e.g. see [18] and [19]. Common to the large majority of these methods is the use of the log-ratio for ranking and classifying probes. In our notation it means sequence identity *Sb *is predicted from array signal *Sa *- *Ra*. This is a paradigm inherited from the analysis of expression data. However, for bCGH data it is actually possible to test how informative this quantity is, since we can perform experiments with one sequenced genome against another. This was done by [6] and [7], and from both publications we may conclude that combining *Sa *and *Ra *in other ways than just subtracting one from the other, is superior. In this paper we have a much larger data set, and the results from Figure 3 clearly show the same picture. For both weak and hard flagging ranking by the bias-corrected S-signal produce larger AUC values than ranking by log-ratio. Hence, we can extract more information from array-signals than just the log-ratios.

In our present approach we have also utilized the sequence information *Rb *directly in the prediction of *Sb*. This seems like a new idea, even if [20] has utilized sequence information in the analysis of single-channel CGH data. When predicting the sequence identity of the S-genome, *Sb*, we first consider how sequence identity *Rb *and array signal *Ra *relates to each other, and then use this to predict *Sb *from *Sa*. The reason a rather obvious approach like this has not be tried out long ago must be due to the tunnel-vision imposed by the log-ratio paradigm. In our approach we treat signals from dual-dye arrays almost as if they were from two single channel arrays, and then use the signal-genotype relation on one array to predict the signal-genotype relation on the other. For this reason the implementation of our method for single channel arrays is straightforward. The only requirement is that for each sample-genome investigated there is also a set of reference signals, i.e. at least one array must be used to hybridize an already sequenced genome to obtain these reference signals.

An argument for using log-ratios is that probe signal biases are canceled. Since we do not use log-ratios, we compensate for this effect by estimating a probe bias from Eq. 4 and then subtract it in Eq. 6. Figure 4 indicates that the weight *ω *should be large, somewhere between 0.7 and 1.0. However, the differences in geometric average are small for various choices of *ω*, and even at *ω *= 0 it is well above 0.9. The values at *ω *= 0 also indicates the precision we get for analyzing a single array, because here we do not borrow any information across arrays. Hence, these results indicates only a small gain in performing a batch of hybridizations, and analyze all arrays together compared to doing it array-by-array.

In Table 2 the results for classification in all 32 data sets are displayed. For the 28 *S. aureus *data sets the picture is clear: Our proposed method, denoted BCSP, performs better than the log-ratio-based mixture model, which is the 'winner' in [4]. For all four criteria sensitivity, specificity, positive predicted value and negative predicted value, the BCSP method gives significant improvement to the mixture model method (small p-values). Noticeable is also the difference between weak and hard flagging. By hard flagging around 1000 ordinary probes are removed from the data set (in addition to all negative control probes), while with weak flagging none are removed. Sensitivity is always improved by hard flagging, but specificity is poorer. The latter means Absent probes become more difficult to detect after hard flagging. This is natural for the BCSP method, since the informative negative controls are no longer available. In general, hard flagging means there are fewer data with small *Ra *and *Rb*-values, and the shape of the functions displayed in Figure 2 become more uncertain and difficult to estimate. Given the excellent results for weak flagging, we can think of no good reason to throw away a large proportion of the probes in a hard flagging procedure. For the *E. faecalis *data the results are more unclear. For weak flagging the BCSP method gives better sensitivity, specificity and NPV, but slightly poorer PPV. No differences are significant, basically because there are only 4 data sets. For hard flagging BCSP produce absolute no specificity, i.e. no truly Absent probes are classified as absent! This illustrates the dramatic effect of losing all information about negative controls and other probes with *Rb*-value equal to 0. Also the mixture model behaves poorly for hard flagging, and again this support a weak flagging strategy.

A difference between the *S. aureus *and *E. faecalis *data is that the *S. aureus *array contain probes representing features in several genomes, a multi-genome array, while the *E. faecalis *array contain little more than what is found in the strain V583. Hence, in the *S. aureus *case there is always a large number of probes that should not hybridize against a specific *S. aureus *genome used for reference. This situation is ideal for our proposed method because there will always be a good balance between probes with small and large *Rb*-values. In a recent publication [21] argues that for multi-genome arrays a mixture of all genomes represented on the array should be used as the reference DNA pool. Their conclusion is based on an analysis of log-ratios. For our supervised learning approach, this strategy should clearly be avoided. If you want to discriminate between Present and Absent in the S-genome channel, you must make certain you have data that show the difference between Present and Absent in the R-genome channel as well. Hence, there should always be a substantial amount of probes against which a reference does not hybridize. Figure 6 illustrate that reliable predictions of sequence identity can only be given for very low or very high identities, i.e. for probes who are either more or less completely Absent or Present. Thus, even if our proposed method opens up the possibility to use and predict any sequence identity, intermediate identities always introduce difficulties. Thus, predicting an identity around 0.5 can be seen as an indication of a large uncertainty.

## Conclusion

We have proposed a method for analyzing bacterial CGH data that seems to be a significant improvement compared to any log-ratio based approach, as indicated by the ROC-analysis. For actual classification we also tend to get improved results compared to the log-ratio based mixture model approach, which was the 'winner' in the survey of [4]. Instead of forming log-ratios, we employ a supervised learning approach where sequence identities are predicted from bias-corrected array signals in each channel separately. The proposed method require a substantial number of probes with little or no sequence identity to the reference genome used in the hybridization. Thus, the method is particulary well suited for data from multi-genome arrays.

## Availability

R code for handling bCGH data using this method, as well as other approaches, is freely available from the corresponding author.

## Authors' contributions

LS has proposed the methods presented, done all the programming in R and drafted the manuscript. OLN has discussed the proposed methods and performed all the *S. aureus *hybridizations. MS has performed the *E. faecalis *hybridizations. ÅA has supplied/constructed the arrays, and been the supervisor of OLN and MS. IFN has been the project leader. All authors have read and approved the final manuscript.
